# Active Cellulose Acetate/Chitosan Composite Films Prepared Using Solution Blow Spinning: Structure and Electrokinetic Properties

**DOI:** 10.3390/polym15153276

**Published:** 2023-08-02

**Authors:** Ana Kramar, Thomas Luxbacher, Nasrin Moshfeghi Far, Javier González-Benito

**Affiliations:** 1 Department of Materials Science and Engineering and Chemical Engineering, Universidad Carlos III de Madrid, Avda. Universidad 30, 28911 Leganés, Spain; 100458500@alumnos.uc3m.es (N.M.F.); javid@ing.uc3m.es (J.G.-B.); 2 Institute of Chemistry and Materials Álvaro Alonso Barba, IQMAAB, Universidad Carlos III de Madrid, Avda. Universidad 30, 28911 Leganés, Spain; 3 Anton Paar GmbH, 8054 Graz, Austria; thomas.luxbacher@anton-paar.com

**Keywords:** cellulose acetate, chitosan, solution blow spinning, protein adsorption, food packaging

## Abstract

Cellulose acetate (CA), a very promising derivative of cellulose, has come into the focus of research due to its highly desired good film-forming ability for food packaging applications. Frequently, this derivative is used in combination with other compounds (polymers, nanoparticles) in order to obtain active materials. Here, we report the preparation of thin films made of cellulose acetate loaded with chitosan (CS) using the solution blow spinning (SBS) method. Films are prepared by SBS processing of the polymers mixture solution, considering the following variables: (i) the concentration of cellulose acetate and chitosan in the solution and (ii) the solvent system consisting of acetic or formic acid. The prepared materials are characterized in terms of physical properties, roughness (optical profilometer), porosity, wettability (contact angle measurements), chemical structure (Fourier transform infrared spectrometry), and electrokinetic properties (zeta potential). SBS enables the preparation of CA/CS films with high water vapor permeability, high porosity, and also higher water contact angle compared with pure CA films. The electrokinetic properties of composites are influenced by the inclusion of chitosan, which causes a shift of the isoelectric point (IEP) towards higher pH values, but the magnitude of the shift is not in correlation with chitosan concentration. Adsorption kinetic studies using bovine serum albumin (BSA) as a model protein reveal that chitosan modified cellulose acetate films manifest low affinity towards proteins that suggests prevention of biofilm formation on its surface.

## 1. Introduction

Food safety and quality can be considered part of the most important aspects of the modern society, being explicit challenges of the United Nations 2030 Agenda for Sustainable Development [1]. Considering that one-third of all food is estimated to be lost or wasted [2], proper storage of food can reduce food waste by extending its shelf-life and preventing food-borne infectious diseases. Because of this, new approaches should focus on finding materials which allow exerting beneficious effects (active materials) on the packed food. Research within the food packaging field is also focused on the use of renewable resources for advanced packaging materials production by following principles of sustainable development and reducing non-biodegradable plastic accumulation [3,4].

Biopolymers are excellent candidates for food applications because they are renewable, eco-friendly, biodegradable, biocompatible, and nontoxic [3,5,6,7]. Cellulose acetate (CA) is one of the most important derivatives of cellulose [8,9]. It is a thermoplastic with a high melting point and excellent film-forming properties; the interest in CA is highly increasing for use in food packaging [8,10,11]. However, like other cellulose-based materials, cellulose acetate is susceptible to microbial growth. Frequently, it is produced with the addition of some active agents that can provide antibacterial properties for the final material [4,12,13,14,15,16]. Having in mind the necessary sustainability, it would be of utmost interest to combine cellulose acetate with antimicrobial biopolymer additives such as chitosan.

Chitosan (CS) is a biopolymeric derivative of chitin found in nature, mostly in the outer shell of sea animals [17,18]. Even though chitosan is currently very expensive, the benefit of using a biodegradable and bioactive polymer alongside cellulose acetate could give a very high added value to the material composite. It is already established that cellulose, its derivatives, and chitosan have good compatibility and can provide antimicrobial materials of wide bioactivity [19,20,21,22,23]. As a consequence, there is an increasing interest in the combination of these two polymers (cellulose acetate and chitosan) for the production of films to be used in food packaging [24,25].

There are, however, some important issues with using natural polysaccharides for food packaging: their poor barrier properties and high wettability [7,26]. This can be addressed, with cellulose acetate as the less hydrophilic derivative of cellulose, by changing its degree of substitution or morphology of the final material [27,28]. On the other hand, considering the hydrophilicity of chitosan, this property is closely related to the chitosan source [29] and the application method. It has been shown that chitosan can bring hydrophobicity to paper [30], either by filling free pores or by interacting with OH groups of the cellulose, thus preventing the OH groups in cellulose from interacting with water.

Polymer-based materials are usually used in the form of films for food packaging. At the laboratory level, because of its ease, the usual method for the preparation of polymeric film is casting [24]. Besides solution casting, electrospinning as a film-forming technique has recently been revived to prepare relatively thin materials based on cellulose with potential use as food packaging films [10,11,14,15,16,31]. Furthermore, electrospinning has also been explored to prepare nonwoven mats of cellulose acetate with chitosan for filtration [32], biomedical applications [33], or as drug delivery systems [34]. However, apart from requiring high electric fields, electrospinning usually implies low productions rates. Therefore, other processing methods should be at least studied with a certain degree of depth.

In this work, we propose a new method, solution blow spinning (SBS), for the preparation of thin films of CA/CS intended for food packaging. Solution blow spinning is a versatile technique for fibers and film formation characterized by high material production rates while using a pressurized gas for material formation [35,36]. Unlike electrospinning, in SBS, the main driving force for polymer solution processing is pressurized gas (commonly air) at the exit of a concentric nozzle, rather than an electric field. Depending on the processing parameters, such as the solution properties, pressure of the gas, and injection rate, it is possible to produce materials with different morphologies ranging from submicrometric fibers to films deposited on a collector [28]. One of the biggest challenges in the use of solution blow spinning for polymeric materials preparation is finding a good solvent for the polymer system to yield a solution with good properties for its processability [37,38]. When working with complex systems such as polymer mixtures, the challenge is greater since the solvent must ensure dissolution of both polymers. The particular case of the CA/CS polymer mixture is very challenging since CS is usually soluble in acidic aqueous solutions and not in organic solvents [39], while CA is easily dissolved in organic solvents and, to some extent, in concentrated acids [40]; at the same time, water is a precipitant for CA, while acetone causes precipitation of CS.

This study is focused on optimizing the conditions for solution blow spinning of the polymer mixture chosen to obtain thin polymeric films. Various spinning conditions were tested during preliminary experiments and optimized according to qualitative criteria of production, which included the speed of production, uninterrupted processing, and ease of film manipulation and separation from the collector.

After the films were prepared, a deep characterization of the films is proposed, including a study of physical and surface properties (such as surface roughness, thickness, and porosity),water vapor barrier properties, and their direct relationship with wettability behavior. Finally, electrokinetic properties were studied to gain insight into the surface charge of the produced films, as well as their protein affinity and adsorption. The surface interaction of the films with proteins such as bovine serum albumin (BSA) can be a good predictor of the propensity of the prepared films for biofilm formation [41,42], which is of great importance for potential food packaging applications. Thus, this article will show how the inclusion of chitosan into the CA matrix, compared with neat CA, aids in protein repellency of film surfaces.

## 2. Materials and Methods

### 2.1. Materials

Cellulose acetate, CA (Sigma−Aldrich Merck, St. Louis, MO, USA) average Mn ~30,000 g/mol, acetyl content 39.8 wt%), and chitosan, CS (Sigma-Aldrich Merck, St. Louis, MO, USA, deacetylated chitin, low molecular weight, zero-shear viscosity measured using Haake Viscotester IQ−Thermo Fisher Scientific, at 25 °C of 1 wt% solution in 2% acetic acid is 1.73 Pa·s, degree of deacetylation, DDA = 66%, according to the method described elsewhere [43,44]), were used as received. All solvents, formic acid FA (Panreac, 85% purity), acetic acid, HAc (Panreac, glacial), acetone (HPLC > 99.9%, Sigma−Aldrich Merck, St. Louis, MO, USA), N,N,dimethylformamide, and DMF (HPLC > 99.9%, Sigma−Aldrich Merck, St. Louis, MO, USA) were used as received without further purification. Chlorotrimethylsilane for the coating of dishes before film casting was from Sigma−Aldrich Merck St. Louis, MO, USA.

KCl (Sigma−Aldrich Merck, St. Louis, MO, USA), KOH (0.1 M, Carl Roth, Karlsruhe, Germany), and HCl (0.1 M, Carl Roth, Karlsruhe, Germany) were used for zeta potential measurements. Bovine serum albumin (BSA), lyophilized powder, ≥96 % (agarose gel electrophoresis) (Sigma−Aldrich, St. Louis, MO, USA) was used for adsorption studies. 

### 2.2. Methods

#### 2.2.1. Preparation of Solutions for SBS

**Figure 1:**
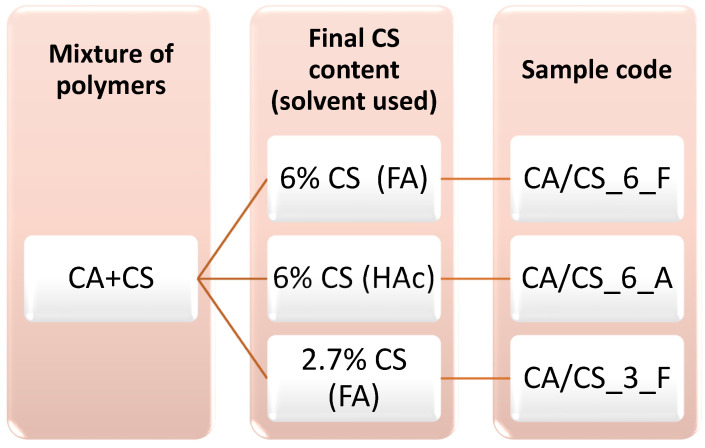
Scheme showing samples code names of the final materials to indicate the proportion of polymers and solvents used to prepare the solutions to be blow spun.

To study the influence of the solvent on the final materials obtained, formic acid and acetic acid were used. Two solutions were prepared using the same proportions of the polymers (8 wt% of cellulose acetate, 0.5 wt% of chitosan) with each solvent, either formic acid (85 wt%) or acetic acid (91 wt%); finally, solid polymer blends of CA/CS with a 6 wt% composition of CS were obtained. On the other hand, the third solution was prepared by dissolving CA 9 wt% and CS 0.25 wt% in formic acid to finally obtain a polymer blends of CA/CS with a 2.7 wt% composition of CS. In Figure 1, as a summary of the preparation of materials by SBS, a scheme is given where the code names of samples used throughout this work are shown. It should be pointed out that the concentrations of CS in solid CA/CS composite materials are calculated from the composition of the polymer solutions used for SBS. Finally, neat CA was produced from a 12 % *w/v* solution of CA in a mixture of acetone/DMF in a 7:3 *v/v* ratio according to a protocol described in the literature [28].

#### 2.2.2. Solution Blow Spinning (SBS) of Porous CA/CS Films

In the solution blow spinning device designed at UC3M [35], a concentric nozzle with an inner channel consisting of a needle protruding 2 mm (inner needle diameter of 0.6 mm) was connected to a high air pressure supply to make the air flow along the outer channel of the nozzle. A pump controlled the injection rate of the polymer solution through the inner channel of the nozzle. The working distance, i.e., the distance between the nozzle and a cylindrical rotating collector, was set at 12 cm. A plastic PE cylindrical collector rotating at 250 rpm was used to collect the materials. For the materials prepared from polymer solutions in formic acid, an air pressure of 1 bar was used; the injection rate of the solution was set at 0.125 mL/min, while for materials produced from polymer solutions in acetic acid, it was possible to increase the injection rate up to 0.25 mL/min with 2 bar of air pressure.

#### 2.2.3. Solution Casting of CA/CS Films

Solution casting onto a glass petri dish was performed with the same solutions of cellulose acetate/chitosan in formic acid or acetic acid simply by leaving the solutions to evaporate under controlled humidity at 35% RH for 72 h. Before casting, the glass was coated with chlorotrimethylsilane to prevent strong adherence of films to the glass surface [45]. The cast films were used to compare the electrokinetic properties of the films prepared by the two different methods: the novel SBS method and the traditional casting method.

### 2.3. Characterization Techniques

#### 2.3.1. Optical Microscopy—Profilometry

The morphology of the SBS films was analyzed using an optical profilometer Olympus DSX500 (Olympus Iberia, Barcelona, Spain). Arithmetic mean roughness (*Ra*) was measured according to standard EN ISO 4288 [46], whereby an average value of *Ra* was calculated from 10 linear profiles (5 in the X direction and 5 in the Y direction) over a surface area of 507 × 507 µm^2^.

#### 2.3.2. Porosity

After the SBS preparation of films, samples were characterized using the following methods:

The thickness of the produced samples was measured using an Easy-check Neurtek Instrument, and the thickness is presented here as the average value of 10 measurements.

The porosity of the produced samples was determined gravimetrically by dividing the density of the sample (*ρ_s_*) with the theoretical density (*ρ_t_*) of the non-porous composite [47], according to Equation (1):

(1) $$\varphi =1-\frac{\rho_{s}}{\rho_{t}}$$

The theoretical density was estimated by applying rules of mixtures [48], according to Equation (2):

(2) $$\rho_{t}=\rho_{f}V_{f}+\rho_{m}V_{m}$$

where *ρ_f_* and *V_f_* are the density and volume fraction of the filler (chitosan), respectively, while *ρ_m_* and *V_m_* are the density and volume fraction of the matrix (cellulose acetate).

#### 2.3.3. Water Vapor Permeability

The water vapor permeability test was performed by a slightly modified procedure of the one described in standard ISO 2528:2017. Test samples were cut into round pieces and placed on top of a vial containing a certain amount of water. Specimens taken from the films were large enough to completely cover the opening of the vial and were closed tightly below the edges of the vial using parafilm. As a positive and negative control, one vial was left completely open and one vial was closed with parafilm, and the controls were tested at the same time as the materials under study. Tests were performed in triplicate, and the results are given as the mass of water per square meter of film per day, expressed as a percentage of water permeability after normalizing the data with the data obtained in the case of using the vial without cover and multiplying by 100.

#### 2.3.4. Wettability—Static Contact Angle Measurement

Static contact angle measurements were performed using the sessile drop method on an OCA-15 Plus Goniometer (Data Physics, Neurtek Instruments, Eibar, Spain). Distilled and deionized water was used as the testing liquid, and photographs were taken after dispensing a drop of 3 µL volume on the surface of the films. The results are expressed as the mean of 5 measurements per sample performed immediately upon contact of the film with water.

#### 2.3.5. Structural Characterization—ATR-FTIR

The investigation of the molecular structure of samples was performed using a Nicolet iS 5 spectrometer (Thermo Fisher Scientific S.L.U, Alcobendas (Madrid) Spain) coupled with an ATR device with a diamond window, GladiATR (PIKE Technologies,). The samples were measured in a range of 400–4000 cm^−1^ using 32 scans and a 4 cm^−1^ resolution.

#### 2.3.6. Surface Charge Measurements and Adsorption Studies

Surface charge was measured using a SurPASS 3 device (Anton Paar, Graz, Austria) in the streaming potential mode. Samples were mounted in an adjustable gap cell, and zeta potential was recorded in an aqueous KCl solution of 1mM ionic strength over a wide range of pH values (from 4.0 to 9.5). Before measurement, the pH of the aqueous KCl solution was adjusted using KOH. Automatic titration was performed using 0.05 M HCl. In all measurements, ultra-pure deionized water was used.

Adsorption studies were performed on the same device using bovine serum albumin (BSA) as a model protein to predict the affinity of the surface towards proteins [49]. In this way, it is possible to predict if proteins can be adsorbed and permanently bonded onto the material’s surface or be repelled from the film’s surface at a particular pH due to electrostatic interactions. Adsorption studies were performed at a pH of 4.5, since this pH value is common for some fresh fruits and vegetables [50]. The adsorption of BSA was investigated for three different concentrations of BSA: 0.02 mg/mL, 0.05 mg/mL, and 0.1 mg/mL. The solutions were prepared by dissolving BSA in 1 mM KCl adjusted to a pH of 4.5.

## 3. Results and Discussion

### 3.1. Morphology and Physical Properties of CA/CS Films

**Figure 2:**
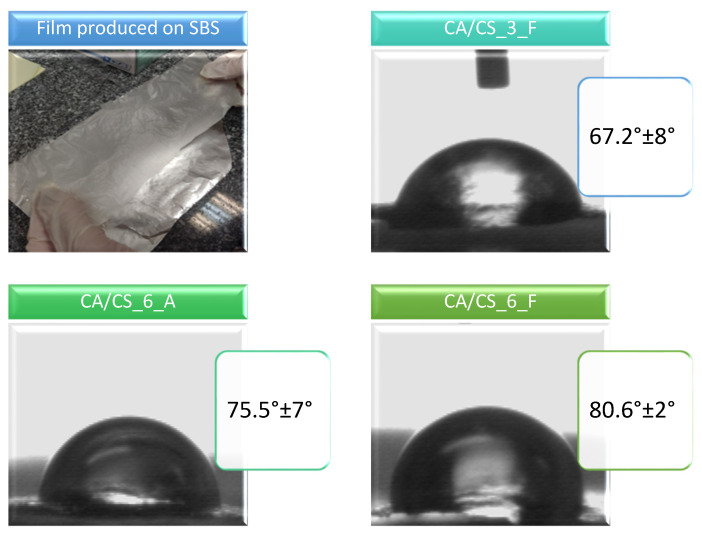
Example of CA/CS film produced using solution blow spinning (size after the removal from the collector 18 × 5 cm^2^); the water drops and corresponding contact angles on films’ surfaces.

Solution blow spinning can be used to produce not only nanofibers, but also flat solid films [28]. In this work, we prepared films of cellulose acetate and chitosan with a size of 18 × 5 cm^2^ using SBS (Figure 2).

**Figure 3:**
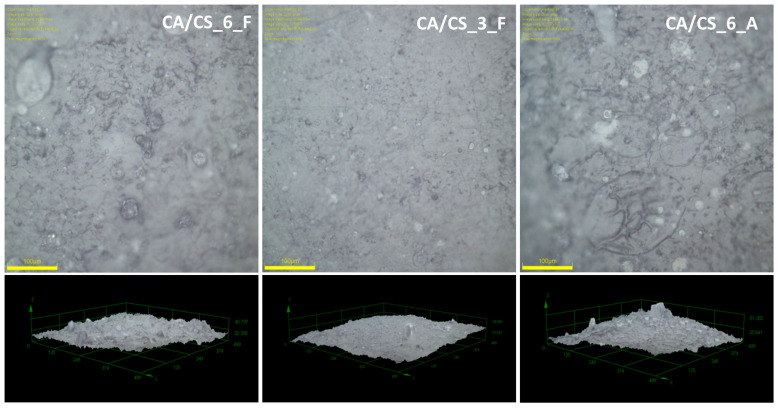
2D (top) and 3D (bottom) optical images of cellulose acetate/chitosan composite films produced using SBS.

When films are observed under the optical microscope (Figure 3), it can be seen that the films are formed by drying droplets of various sizes mutually connected into a more complex structure. The average surface roughness is between 2 and 3 µm. It seems that a lower chitosan content induces a slightly lower surface roughness (Table 1), while CA samples with 6 wt% of chitosan have the same surface roughness regardless of the conditions of SBS processing. The produced films also exhibit high porosity and high water vapor permeability (Table 1); these properties can be beneficial for food packaging, especially for fresh food such as fruits or vegetables, where condensation and increased moisture can accelerate the deterioration of food and promote the growth of fungi [50].

On the other hand, films with higher chitosan contents (6 wt%) have higher contact angles (i.e., lower wettability), implicating that higher concentrations of chitosan must improve the performance of these materials in terms of protection from water. It can be concluded that the addition of higher contents of chitosan, even though chitosan itself is hydrophilic, induces higher barrier properties toward water; this is because an increase of film roughness causes morphology variations, leading to films that are more suitable for potential food packaging. At this point, it is important to highlight the influence of the preparation route of these CA/CS composites. As stated in earlier works, CA is a hydrophilic polymer which, in the form of cast films, usually exhibits a contact angle in the range of 60–70° [24,45,51], and the addition of chitosan can further reduce the contact angle, making the final material very hydrophilic [24]. In another work by our group, it was shown that CA films prepared by SBS can result in various levels of hydrophobicity, depending on the morphology [28]. For example, in the case of a flat CA film prepared with SBS, a water contact angle of approximately 69° was obtained [28]. In this current work, the addition of chitosan reduces wettability, possibly due to the increased surface roughness in a highly porous film; this allows the Cassie–Baxter state to be dominant [52] as a consequence of having more voids and pores with air incorporated in the film, contributing to the observed hydrophobic behavior [28,53].

### 3.2. Structural Characterization of CA/CS Films

To study the molecular interaction between cellulose acetate and chitosan, ATR-FTIR spectra were analyzed.

**Figure 4:**
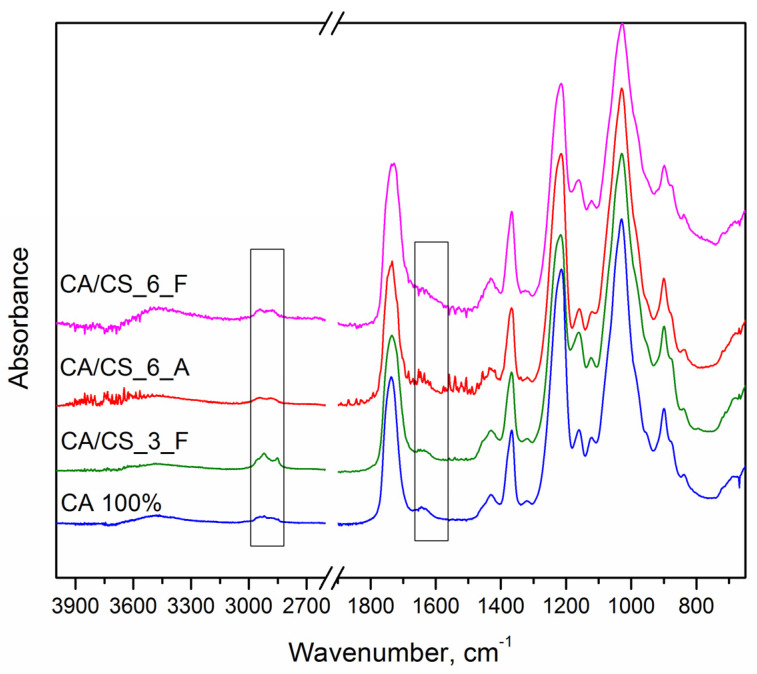
ATR−FTIR spectra of 100% CA film and CA/CS composite films prepared using various concentrations of CS and solvents (A−acetic acid, F−formic acid) during SBS: marked regions correspond to prominent changes due to inclusion of CS into CA.

As can be seen in Figure 4, all spectra show the typical absorption bands of cellulose acetate. The broad weak band around 3490 cm^−1^ corresponds to non-esterified hydroxyl groups (OH stretching) of cellulose; the weak bands at 2945 cm^−1^ and 2886 cm^−1^ are associated with the CH antisymmetric and symmetric stretching of the methyl group, CH_3_, respectively [11,45,54]. The high-intensity absorption band at 1735 cm^−1^ corresponds to carbonyl stretching in the acetyl group, as expected for cellulose acetate, which does not change upon the addition of chitosan. The peaks at 1365 cm^−1^ and 900 cm^−1^ assigned to the symmetric CH_3_ bending and to the acetate methyl groups, respectively, are also typical for cellulose acetate [45,54].

Upon the addition of chitosan, there is a change in the spectra in the range of 1550–1650 cm^−1^ (Figure 4). Specifically, the peaks at 1654 cm^−1^, 1590 cm^−1^, and 1560 cm^−1^ are assigned to amide I, N–H bending vibrations of amide II, and stretching vibrations of amino groups, respectively [49,55]. These peaks correspond to the area for which chitosan–cellulose interactions have been reported [56,57]. However, due to the differences between cellulose and cellulose acetate, these interactions are probably less pronounced. When pure cellulose is considered, interactions with chitosan are usually limited to hydrogen bonding and weak dipole–dipole interactions through the OH groups in cellulose [57]. In the case of cellulose acetate, the most probable interactions are between amino groups of chitosan, and acetate groups and a small amount of non-acetylated OH groups in CA [24]. Additionaly, in the spectral range of 2875-2945 cm^−1^, there is also a change of intensity leading to better resolved peaks when CA is modified with the addition of CS. Especially evident is the rise of peaks upon the addition of 2.7 wt% CS, where there is a differentiation of the peak at 2921 cm^−1^, which arises from chitosan aliphatic CH stretchings [58,59].

Having in mind that a lot of similarities in the structure exist between cellulose acetate and chitosan, ATR-FTIR analysis and its interpretation can only focus on qualitative aspects; whereby, the most prominent peaks specific to chitosan (amino groups) should be definitive for the analysis of molecular interactions and material structure. In our work, the characteristic absorption bands of chitosan centered around 1600 cm^−1^ become prominent. For further analysis of possible specific interactions between CA and CS, the zeta potential is presented in the next section.

### 3.3. Surface Charge Analysis of CA/CS Films

**Figure 5:**
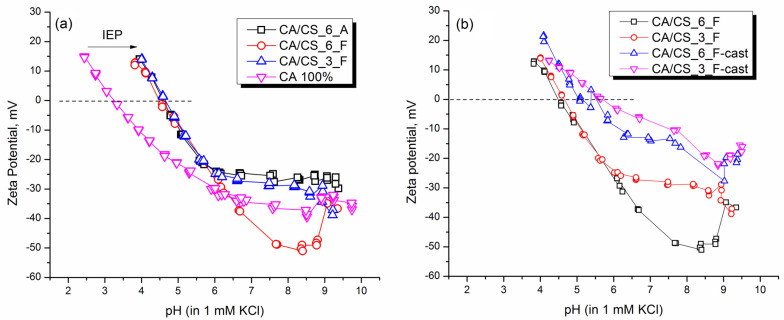
(**a**) Zeta potential measured in the entire range of pH in an electrolyte solution containing 1 mM KCl, for pure CA− and CS−modified CA films produced using SBS, (**b**) comparison of zeta potential between the SBS and cast films with the same compositions.

Surface charge analysis can be very useful, especially when it is necessary to anticipate certain properties of surfaces in contact with liquids. As was mentioned in the Introduction section, active films for food packaging need to have several demands satisfied: for instance, having good liquid barrier properties and frequent antimicrobial action. Chitosan is known to have antimicrobial properties, and, more importantly, it has a positive surface charge over a wide pH range. In fact, this positive charge is considered to be one of the main reasons for the antimicrobial activity of chitosan [60,61]. For example, in the case of bacteria, it is considered that chitosan interacts with the cell walls, thus disrupting their functions and producing a biocide effect [34]. This is why the surface charge of the materials prepared in this work was assessed by the measurement of the zeta potential within a wide range of pH values, ranging from 4 to 9.5 (Figure 5a). The measurements below pH 4 were not considered because chitosan dissolves in acidic environments. As can be seen, compared to neat CA (produced from a 12 % solution of CA in acetone/DMF), in which the IEP was detected at pH 3.25, the addition of chitosan causes a shift of the isoelectric point to a value of pH between 4.5 and 5; whereby, there is no correlation between the content of chitosan and the pH of IEPs. In this work, the addition of either 2.7 wt% or 6 wt% of chitosan to cellulose acetate produced the same effect regarding the IEP shift toward higher pHs. However, considering the cast films (Figure 5b) prepared using the same solutions as in the case of SBS, it can be concluded that casting as a method of film production causes a slightly higher pH of the IEP (pH 5.0 and pH 5.5 for films cast from formic acid solutions that contain 6% and 2.7% of chitosan, respectively). This means that besides the content of chitosan, the method of preparation of films is important and can influence the IEP of the films. Also, a lower amount of chitosan seems to induce a greater effect on the IEP shift, which is probably a consequence of a better dispersion of chitosan in the CA matrix. Since solution blow spinning is demonstrated to be a good method to achieve uniform dispersion of fillers into polymer matrices [62,63], when using this processing method, a quite uniform dispersion of chitosan within the CA matrix is expected; however, when a casting method is used to prepare the materials, there might be greater accumulation of chitosan near the surface, which induces higher IEPs.

Considering specific interactions between CA and CS, it is also possible that in SBS films, besides having better dispersion of chitosan in the CA matrix, there is better interaction of amino groups of chitosan with acetate and hydroxyl groups in CA; therefore, they do not contribute to the positive surface charge of composite films to a higher extent. This is further corroborated with the fact that the addition of CS does not produce a further shift of the IEP, even though pure CS has an IEP of 7.4.

**Figure 6:**
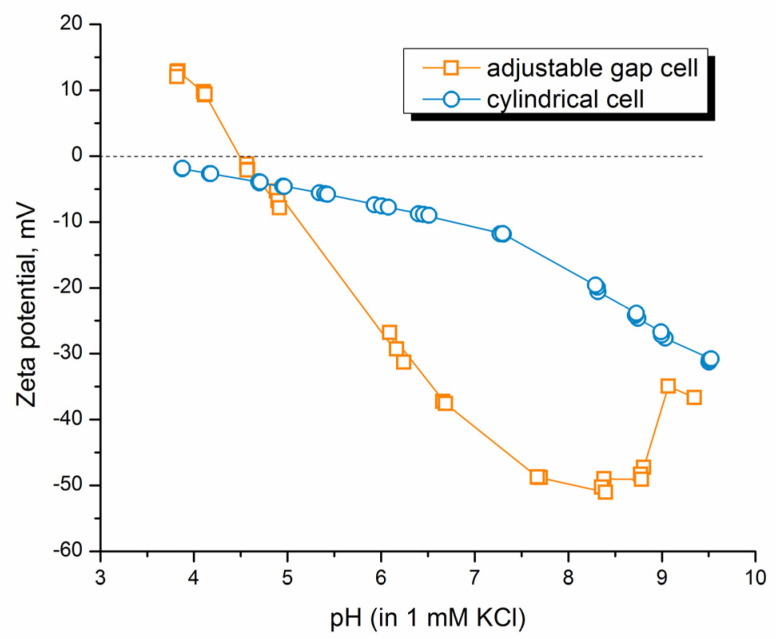
Comparison of zeta potential curves of the CA/CS_6_F sample measured in adjustable gap cell and cylindrical cell.

Because films produced with SBS are porous, we carried out the measurements of zeta potential in two different cells: in an adjustable gap cell, where the electrolyte flows over the sample surface (tangential flow of the electrolyte) and a cylindrical cell, where the liquid flows through the porous film (Figure 6). As can be seen, when using an adjustable gap cell, the IEP of the CA/CS_6_F sample is detected at a pH of 4.5, higher than the one obtained for neat CA. Furthermore, when the same film is measured in the cylindrical cell, a completely different curve that is not even able to reach the IEP is obtained.

Below pH 7, there is a continuous increase of the zeta potential, ζ, until it seems to level off at about pH = 4; although, the zeta value ζ = 0 mV is not reached. Therefore, it can be concluded that when using a cylindrical cell, the electrolyte solution passes through the film, which contains a high ratio of CA compared to CS, without it being possible to detect the positive charge of chitosan. Another important thing to mention is that the curve of zeta potential obtained when using a cylindrical cell is typical of that obtained for swelling processes of materials (low zeta potential closer to 0 mV and inability to reach the IEP [64]); therefore, intra-flow of the electrolyte in the systems under study causes greater swelling than tangential flow over their surface.

### 3.4. Adsorption Studies of Bovine Serum Albumin (BSA) onto CA/CS Films

**Figure 7:**
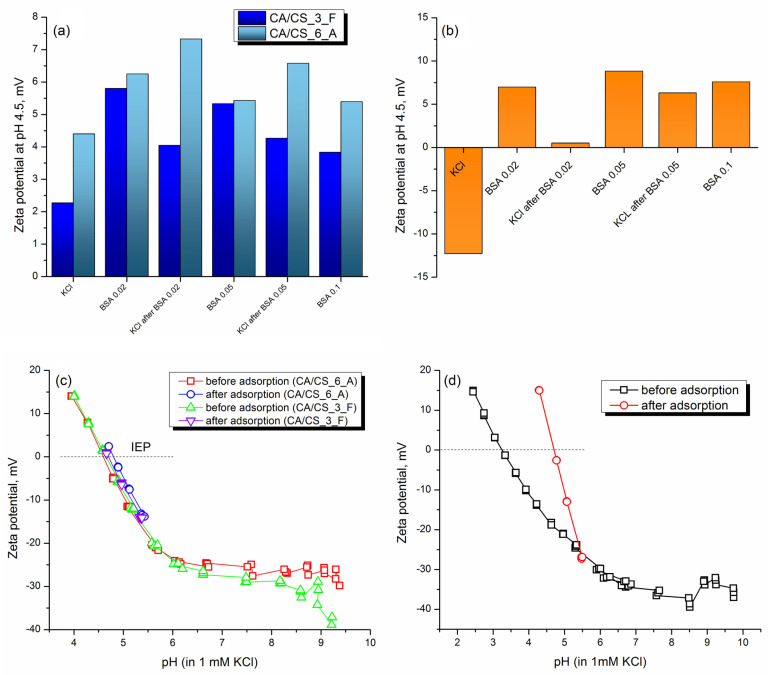
(**a**) Zeta potential at pH 4.5 before and after adsorption of various concentrations of BSA (0.02, 0.05, and 0.1 mg/mL) onto CA/CS films produced using SBS; (**b**) zeta potential at pH 4.5 of neat CA film produced using SBS before and after adsorption of BSA (0.02, 0.05 and 0.1 mg/mL); (**c**) zeta potential of CA/CS films in the entire pH range and isoelectric points (IEPs) detected before and after the adsorption and (**d**) zeta potential of CA film before and after the adsorption of BSA.

Biofilm formation on material surfaces is usually related to uncontrolled protein adsorption [41,42]. To study the protein repellency of CA/CS films, we have performed adsorption studies using BSA as a model protein. For protein-repellent surfaces, it is considered that they can prevent biofilm growth [41,42]. The adsorption studies were carried out using several steps: (i) zeta potential measurement at pH 4.5 in an aqueous solution of 1 mM KCl, (ii) adsorption of BSA, (iii) zeta potential measurement in BSA solution, (iv) two cycles of rinsing with KCl and three cycles of rinsing with KCl set at pH 4.5, (v) measurement of zeta potential in 1 mM KCl at pH 4.5 after the adsorption. This protocol was performed sequentially for all investigated BSA concentrations. The results are shown in Figure 7.

As can be seen, CA/CS composite films already have a positive charge before adsorption experiments (Figure 7a), and after adsorption, there is a small increase of zeta potential in a range of around 2 mV. After the adsorption of the maximum concentration of BSA used in this work (0.1 mg/mL), a decrease in zeta potential is obtained, compared to the previous step (adsorption of 0.05 mg/mL). This result is probably a consequence of the further repulsion of positively charged BSA and the positively charged surface of the composite film with small amount of BSA adsorbed. The isoelectric point of BSA is approximately at pH 4.9 [65,66].

**Figure 8:**
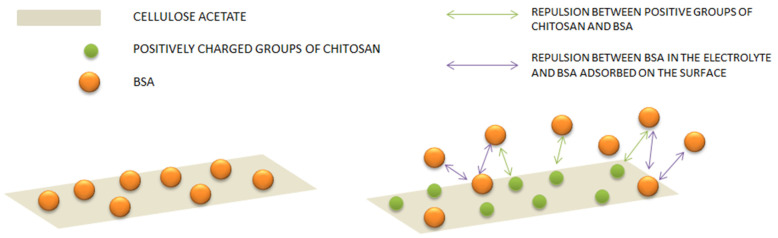
Scheme representing the possible interactions between bovine serum albumin (BSA) and surface of neat cellulose acetate and film functionalized with chitosan during the adsorption studies.

After the first adsorption step (using BSA solution of 0.02 mg/mL) and after a rinsing cycle, the zeta potential in 1 mM KCl at pH 4.5 had increased values of only 1.8 and 2.9 mV in CA/CS film containing 2.7 wt% and 6 wt% of chitosan, respectively. Considering the increase of 12.7 mV after the first adsorption of BSA and rinsing from neat cellulose acetate film, the increase in CA/CS composite films is very low. The further increase in BSA concentration studied for adsorption (to 0.05 and 0.1 mg/mL) revealed that there is a further decrease in zeta potential in composite films. It seems that there exists a very complex electrostatic interaction between charged BSA and CA/CS surfaces, and there is possibly an interaction between BSA particles as well (Figure 8). The zeta potential of CA films prepared using SBS without chitosan (Figure 7b) is significantly different after the adsorption of BSA compared with CA/CS films. In this case, after the first low-concentration adsorption of BSA, there is a shift of zeta potential at pH 4.5 of almost 20 mV. After rinsing, there is a slight decrease of zeta potential, but it remains in the positive range, above 0 mV, indicating significant electrostatically firm interactions between the surface of the CA film and BSA, as can be depicted in the scheme in Figure 8. A further increase in the concentration of adsorbed BSA leads to an increase in zeta potential, reaching a final value of 7.5 mV at pH 4.5 even after rinsing, and a shift of the IEP in neat CA from pH 3.25 to pH 4.7, indicating permanent binding of BSA to the CA surface, since the resulting IEP is one close to the IEP of BSA [66].

In the case of CA/CS films, the final curve of zeta potential after adsorption and rinsing (Figure 7c) reveals that there is no significant shift after the adsorption experiments, indicating the stability of the surface of CA/CS composite films and their resistance toward significant protein adsorption. The zeta potential at pH 4.5 of CA/CS films after final adsorption and rinsing is between 1 and 2.4 mV. Unlike CA/CS, pure CA film (Figure 7d) obviously has a significant affinity towards proteins like BSA, indicating a strong electrostatic attraction between CA films and proteins. Therefore, in terms of potential protein repellency and lower protein affinity, the modification of CA with CS and their processing into a composite film by solution blow spinning are very efficient.

## 4. Conclusions

In this work, we present, for the first time, the preparation of composite cellulose acetate/chitosan films using solution blow spinning (SBS). Solution blow spinning as a film processing technique has a lot of potential for the preparation of composite films, especially from biopolymers such as cellulose acetate and chitosan. The prepared composites are highly porous films that are formed by coalescing microdroplets on the collector during SBS. These composite films can be prepared either from acetic acid or formic acid solutions of the polymer mixture. CA/CS composites exhibit high water vapor permeability (up to 78.5% water vapor can pass through them compared with the open vial); at the same time, due to their high porosity and existence of voids and air pockets, they exhibit a higher contact angle with water during wetting compared with neat CA films prepared in SBS. Surface charge was measured with the streaming potential method, and the results confirm that the inclusion of chitosan induces a shift of the isoelectric point. Below pH 4.5 films have a positive surface charge, and they show lower protein affinity compared with neat CA; this was analyzed through adsorption studies at pH 4.5 of the protein bovine serum albumin, BSA, in several concentrations (0.02, 0.05, and 0.1 mg/mL) onto composite films. The good protein repellency and low protein affinity can prevent biofilm formation on films’ surfaces; considering its high water vapor permeability but also lower wettability (which can prevent moisture buildup in the packaging of, e.g., fresh fruit), it can be concluded that CA/CS composite films can be suitable as a potential candidate for food packaging applications.

## Figures and Tables

**Table 1:** Some physical properties and water vapor permeability of cellulose acetate/chitosan composite films prepared using solution blow spinning.

Sample Code	Surface Roughness (*Ra*), µm	Porosity, %	Water Vapor Permeability, %
CA/CS_3_F	1.9 ± 0.4	63	78.5
CA/CS_6_F	3.2 ± 0.5	58	76.3
CA/CS_6_A	3.2 ± 0.6	62	76.7

## Data Availability

The data presented in this study are available on request from the corresponding author.
